# Improving the Innate Immune Response in Diabetes by Modifying the Renin Angiotensin System

**DOI:** 10.3389/fimmu.2019.02885

**Published:** 2019-12-10

**Authors:** Maira Soto, Kevin J. Gaffney, Kathleen E. Rodgers

**Affiliations:** Pharmacology Department, College of Medicine, Center for Innovation in Brain Science, University of Arizona, Tucson, AZ, United States

**Keywords:** diabetes, immunosuppression, innate, PMNs, alveolar-macrophages, angiotensin, NorLeu

## Abstract

Patients with Type 2 Diabetes Mellitus (T2DM) suffer from a higher incidence and severity of pulmonary infections. This is likely due to immune impairment and structural abnormalities caused by T2DM-induced oxidative stress (OS) and chronic inflammation. Modulation of the Renin Angiotensin System (RAS) through blockade of the actions of angiotensin II (AII), or inducing the protective pathway, has the potential to reduce these pathological pathways. The effects of Angiotensin 1–7 [A(1-7)] and NorLeu^3^-A(1-7) [NorLeu], ligands of the protective RAS, on the innate immune response were evaluated in the *db/db* mouse model of T2DM. Only NorLeu treatment reduced the structural pathologies in the lung caused by T2DM. A decreased in bactericidal activity and phagocytosis in diabetic animals was also observed; both A(1-7) and NorLeu treatment restored these functions. Myeloid progenitor CFUs were reduced and neutrophil/progenitor OS was increased in saline-treated *db/db* mice, and was reversed by A(1-7) and NorLeu treatment. These results demonstrate the adverse effects of diabetes on factors that contribute to pulmonary infections and the therapeutic potential of protective RAS peptides. Overall, RAS-modification may be a viable therapeutic target to treat diabetic complications that are not addressed by glucose lowering drugs.

## Introduction

T2DM is a worldwide epidemic. In the United States alone, 23.2 million people (7.5% of the population) are currently diagnosed with T2DM. T2DM is now the seventh leading cause of death (1). The collective impact of T2MD-associated complications results in both reduced quality of life and increased economic burden (2).

In addition to the well-recognized complications and co-morbidities associated with T2DM, diabetic patients also suffer from less-studied complications such as decreased lung function and impaired immune function (3–7). Diabetic patients are more likely to suffer from infection and at a greater risk of complications after infection (8–12). During the 2009 H1N1 Influenza pandemic, diabetic patients had a higher rate of hospitalizations (13–15). Diabetes is also linked to higher rates of Methicillin-resistant *Staphylococcus aureus* (MRSA) infection (7). Epidemiological data links diabetes to higher incidence of a variety of cancers, including liver, pancreas and lymphoma; perhaps due to immune suppression (1). Mouse models of hind paw infection show diminished innate immunity at the site of infection and reduced circulating polymorphonuclear leukocytes (PMN) function in diabetic mice (3). PMN counts can be affected by metabolic parameters such as age, BMI, and systolic blood pressure (16). Increases in the frequency of all these ailments in diabetic patients and diabetic mouse models indicate there is a decrease in the activity of the cells that are involved in innate immunity. Dysregulation of PMNs is associated with several diabetic complications, such as hypofibrinolysis, nephropathy and cardiovascular events (17–19). Immune-suppression in diabetic patients happens despite the availability of current glucose control medications for T2DM, highlighting a need for additional therapeutic intervention.

Traditionally, the Renin Angiotensin System (RAS) is known for its role in blood pressure regulation. Both angiotensin II (AII) and angiotensin (1-7) [A(1-7)] are bioactive peptides of RAS; both of these peptides have now been associated with physiological functions that reach beyond the regulation of hypertension. Activation of the angiotensin type I (AT1) receptor by AII results in a number of pathological processes including vasoconstriction, increased pro-inflammatory response, elevated levels of oxidative stress (OS), insulin resistance, hypertension (HTN), and end organ failure (9–12, 20). A(1-7), acting primarily through Mas receptor activation, causes vasodilation, decreased OS and has anti-inflammatory effects (4, 6, 13). These actions of the protective RAS may reduce co-morbidities related to T2DM. The discovery of these protective effects by RAS-modifying peptides has prompted therapeutic interest in this system. NorLeu^3^-A(1-7) [NorLeu], a peptide analog of A(1-7), has already shown efficacy in diabetic wound repair (21–25).

Studies reported herein were designed to further understand the impact of T2DM and RAS modification on immune parameters that are important in clearing respiratory infections, using *Staphylococcus aureus* (*S. aureus*) as a pathogenic model in *db/db* mice. The primary mode of *S. aureus* clearance in this pneumonia model is through alveolar macrophages and neutrophils (26, 27), both main players in pulmonary innate immunity. As chronic inflammation and OS may contribute to the potential immunosuppression in diabetics, A(1-7) and NorLeu were used as novel treatments to correct diabetes-induced immune dysfunction in the *db/db* model.

## Materials and Methods

### Animal Procedures

Male BKS.Cg-Dock7^m^+/+ Lepr^db^/J (*db/db*) mice and age-matched non-diabetic heterozygous controls (*htz*) were purchased from Jackson Laboratories (Bar Harbor, ME, USA). Eight week old mice were randomized into three treatment groups (*n* = 6–10/group). Animals were administered either saline (*htz* and *db/db*), A(1-7) (0.5 mg/kg/day) (*db/db*), or NorLeu (0.5 mg/kg/day) (*db/db*) subcutaneously, daily for 4, 8, or 12 weeks. Dose finding studies previously performed indicated 0.5 mg/kg/day is optimal (28). Pharmaceutical grade A(1-7) and NorLeu were purchased from Bachem (Torrance, CA, USA). Mice were kept on a 12 h light/dark cycle and food and water were available *ad libitum*. Body weight was assessed at necropsy. Blood glucose level was measured using a Free Style Lite meter (Abbott Laboratories, Abbott Park, IL) from a drop of blood obtained from the saphenous vein. All animal studies have been reviewed and approved by the University of Southern California Institutional Animal Care and Use Committee (IACUC).

### Micro-CT Scanning and Analysis

The Inveon preclinical CT scanner (Siemens, Knoxville, TN) was used for data-acquisition in prone position under 2% isoflurane inhalation anesthesia (tube voltage 80 kV, tube current 500 μA, 0.104 mm effective pixel size, binning of 2 to reduce noise) with and without respiratory gating (i.e., synchronization of acquisition of micro-CT projections with a time-point in the respiratory cycle of the individual mouse). Scanning took 10 and 30 min with and without respiratory gating, respectively. Respiratory monitoring was performed using a pressure transducer pad (System BioVet™ ©m2m Imaging Corp, Newark, USA) placed under the animal's chest. Images were reconstructed and assessed at a constant window width/window level (5000/2000). The acquired images were reconstructed using the Inveon™ Acquisition Workplace software (Siemens, Knoxville, TN). A Feldkamp algorithm with a Shepp and Logan filter was used to reconstruct the acquired images. The images were output and stored in a dicom format. The CT image data were analyzed using AMIRA (FEI, Visualization Sciences Group, Houston, TX) to create volume renderings. 3-D segmentation of lung tissue and airway was performed based on gray value threshold difference between tissue and air. Volumes of different lung segments at both inhalation and expiration were automatically quantified by AMIRA. A ratio of inhalation and expiration volumes was used as indirect marker for lung compliance.

### *S. aureus* Survival Assay

*S. aureus* strain Newman was provided by Dr. Annie Wong-Beringer's laboratory (USC). After 6-weeks of treatment, blood was collected from the tail-vein and placed in microvette heparin coated tubes. A 1:10 bacterial solution was prepared, incubated with agitation for 30 min at 37°C and then diluted into fresh DMEM+5% fetal bovine serum (FBS) at 1:12. For each animal, 25μL of blood diluted into 162.5 μL *S. aureus* (6 × 10^6^ CFUs) preparation and incubated at 37°C in a rotating platform for 30 min. Surviving *S. aureus* titers were determined by plating serial dilutions in duplicate on tryptic soy agar (TSA) plates with 5% sheep blood.

### Neutrophil Activity Assays

Twenty microliters of blood were collected into a heparinized tube from the mouse tail vein, the RBCs were lysed and remaining cells were washed and suspended in DMEM+5%FBS. Samples were placed on a 96-well flat bottom plate and 100 μL of 1 mg/mL pHrodo Red *S. aureus* BioParticles Conjugate (pHrodo) in DMEM+5%FBS and 0.5 μL of CellROX OS Reagents (Thermo Fisher Scientific) were added. The *S. aureus* were added in excess to capture maximal phagocytic capabilities. The plate was read at 10 min intervals for 110 min at Ex 509/Em 533 (pHrodo) and Ex 640/Em 665 (CellROX) on a Synergy H1 Hybrid Multi-Mode Microplate Reader (BioTek, Winooski, VT). These samples were also read on a LSR II flow cytometer (BD Biosciences, San Jose, CA). Data were analyzed using FlowJo V 10.0.7r2.

### Neutrophil Phagocytosis Assay

Heparinized blood was collected from the tail vein of treated mice, and total WBCs were counted. Twenty microliters of whole blood were diluted 1:5 with DMEM+5%FBS, 5 μL of 1 mg/mL pHrodo was added and incubated at 37°C for 90 min. RBCs were lysed, the sample was washed and then fixed with 10% buffered formalin. Samples were read on a LSR II flow cytometer. Data were analyzed using FlowJo V 10.0.7r2 to determine the total concentration of PMNs and phagocytic PMNs in whole blood.

### Plasma Collection

At the necropsy, mice were overdosed with ketamine/xylazine and the blood was collected by cardiac puncture into EDTA-coated tubes. Immediately after collection plasma was isolated by centrifugation; plasma aliquots were stored at −80°C for later analysis.

#### Plasma Cytokine Measurements

Plasma collected at necropsy was also used to measure circulating cytokine levels using the V-PLEX Proinflammatory Panel 1 Mouse Kit (Meso Scale Diagnostics; Rockville, MD).

### Bone Marrow (BM) Collection and Cultures

Femurs from un-infected mice were collected after 4, 8, and 12 weeks of treatment and BM was harvested by flushing with PBS containing 2%FBS/2x Pen/Strep. BM cells were washed, counted and re-suspended in DMEM+5%FBS at 5 × 10^6^ cells/mL Mouse MethoCult™ media (StemCell Technologies, Cambridge, MA), 900 μL, was added 24-well tissue culture plates, 20 μL of cells mixed in and cultures incubated at 37°C, 5% CO_2_ in air. After 12 days of culture, CFU-granulocyte, erythrocyte, macrophage and megakaryocyte (GEMM) and CFU- granulocyte macrophage (GM) colonies were counted under phase contrast microscopy.

### 3-Nitrotyrosine (N-Tyr) Staining

BM cells were fixed with 4% paraformaldehyde, incubated with permeabilizing solution (0.1% Triton-X in PBS) for 15 min in the dark at room temperature, and washed. BM cells in 100 μL of 2% FBS in PBS were stained with 1 μL L anti-N-Tyrosine-FITC antibody (EMD Millipore Corp, Temecula, CA; Clone 1A6) 30 min at RT, washed, resuspended in 0.5 mL of 1% paraformaldehyde and stored in the dark at 4°C until flow cytometric analysis. Percentage of PMNs, N-Tyr+ cells and N-Tyr+ PMNs were calculated from this analysis.

### Histological Analysis

At the necropsy lungs were rapidly excised and weighed. The right lobe was formalin-fixed and paraffin-embedded, cut at 7 μm and stained with Hematoxylin and Eosin. Four to five images per slide were taken at 40x magnification, 10 pictures/slide, to count alveolar macrophages.

### *In vitro* Macrophage Function

Raw 264.7 cells were obtained from the ATCC (Manassas, VA). The cells were preincubated for 48 hr at 37°C and 5% CO_2_ under: a. Normal glucose (NG), in DMEM; b. Diabetic milieu (DM) as previously described (29); c. DM + AII (100 nM); d. DM + AII (Bachem) (100 nM) and A(1-7) (100 nM); e. DM + AII (100 nM) and AVE 0991 (MedChem Express, Princeton, NJ) (10 nM); f. DM + AII (100 nM) and AVE 0991 (100 nM); g. DM + AII (100 nM) and AVE 0991 (1000 nM). Next, the cells were harvested, incubated for 1 h with pHrodo and fixed with 4% formaldehyde solution. Mean fluorescence intensity was determined by flow cytometry using a BD™ LSR II flow cytometer.

### Statistical Analysis

GraphPad Prism version 6.0c for Mac OS X (GraphPad Software, San Diego, CA, USA) was used to analyze the data. One-way ANOVA followed by Dunnett's multiple comparisons test were used to compare data. The level of statistical significance was set at 5%. Data are expressed as mean value ± standard error of the mean (SEM).

## Results

### The Overall Diabetic Phenotype of *db/db* Mice Is Not Altered by A(1-7) or NorLeu Treatment for 8 Weeks

BKS.Cg-Dock7^m^+/+ Lepr^db^/J (*db/db*) mice develop very severe diabetic phenotype due to overnutrition, closely resembling human disease, which make it a useful tool for studying potential therapeutics (30, 31). Mouse weights taken at necropsy show that there is no significant weight reduction after treatment in *db/db* mice (Supplemental Figure 1A). Plasma collected at necropsy after 8 weeks of treatment was used to measure circulating cytokines (TNF-α, IL-6, and IL-10) using a multiplex immune-assay. While changes have been noted in these markers with long term (16 weeks) A(1-7) treatment in this model, no significant changes were detected in any of these groups with 8 weeks of treatment (Supplemental Figure 1B) (29).

### Diabetic Animals Show Reduced Lung Size, Air Volume Capacity, and Air Volume at Inhalation/Exhalation Ratios, Despite Treatment

**Figure 1:**
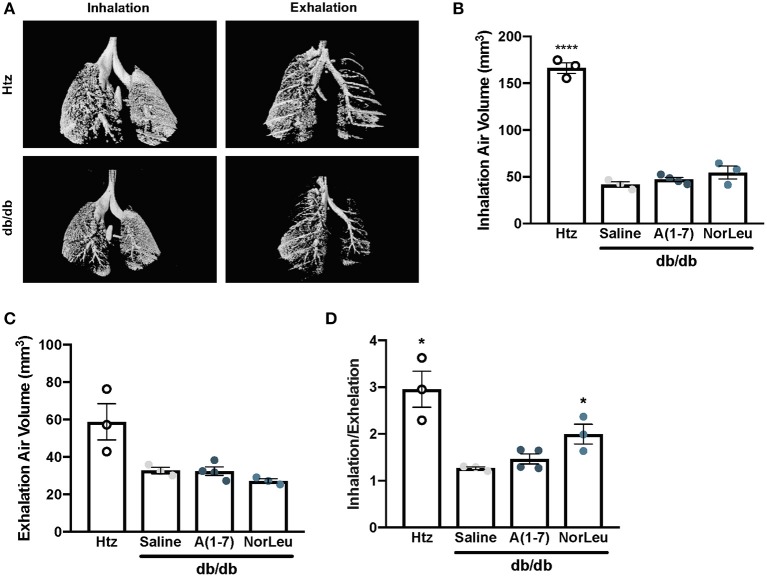
Lung capacity is significantly reduced in diabetic mice. Diabetic mice were treated with saline, A(1-7) at 2 mg/kg/day, or NorLeu at 2 mg/kg/day for 8 weeks starting at 8 weeks of age. microCT-scans were used to visualize air volume in the lungs of treated mice during normal inhalation and exhalation and compared to htz saline treated mice of the same age **(A)**. Air volume at inhalation **(B)** and exhalation **(C)** was calculated based on 3D pixel density. A ratio of inhalation/exhalation was calculated to estimate lung elasticity **(D)**. Statistics was done using Prism 6 software ANOVA and compared to saline treated *db/db* mice; ^*^p ≤ 0.05, ^****^p ≤ 0.0001.

Micro-CT scans of mouse lungs can give insights to the global environment of the lung. In 16-week-old control *db/db* and *htz* mice, images of the lung were taken at inhalation and exhalation (Figure 1A). Air volume in the lungs at each measurement was quantified. Consistently, 16-week-old *db/db* animals had lower % air volume during inhalation and exhalation (Figures 1B,C), even when corrected for total body weight of the mouse. The inhaled/exhaled air volume ratio can be used as a marker for lung elasticity and is also significantly lower in the diabetic animals treated with saline (Figure 1D). A(1-7) treatment—started at 8 weeks of age and continued for 8 weeks—had no effect on this ratio. However, mice treated with NorLeu had a significant improvement in inhalation/exhalation ratios. Although both A(1-7) and NorLeu are thought to target the same receptor, NorLeu has previously shown to have pronounced anti-fibrotic effects beyond those observed after A(1-7) treatments (32).

### Diabetic Mice Show Diminished Pathogen Clearance in *ex-vivo* Neutralization Assays of *S. aureus* While A(1-7) and NorLeu-Treated Diabetic Mice Did Not

**Figure 2:**
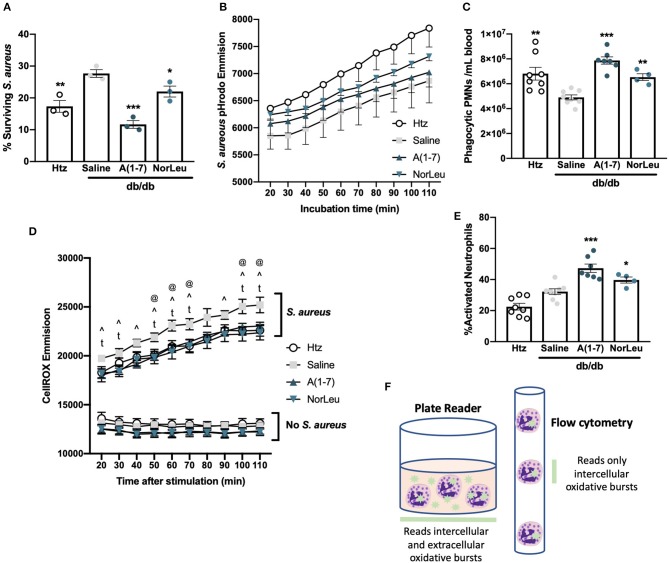
PMNs from *db/db* mice favor degranulation in response to bacterial challenge and those from A(1-7) treated mice favor phagocytosis. Tail-vein blood was collected from htz and *db/db* mice treated with saline or A(1-7) at 0.5 mg/kg/day. Whole blood was incubated with live *S. aureus* for 30 min and the percentage of surviving *S. aureus*-CFUs was calculated **(A)**. Whole blood samples were also incubated with *S. aureus*-pHrodo; kinetics of phagocytosis were measured using a plate reader with no significant changes between groups at any time period **(B)**, and number of phagocytic PMNs after 90 min **(C)** was measured using flow cytometry. Blood samples were also incubated with CellROX in the absence and presence of *S. aureus*, the emission was measured with a plate reader for 90 min starting at 20 min at 10 min intervals **(D)** There were only significant differences between treatment groups with *S. aureus* where saline treated *db/db* mice were: significantly higher than *htz* at 50, 60, 70, 100, and 110 min (^@^); significantly higher than A(1-7) at 20, 30, 40, 50, 60, 70, 90, 100, and 110 min (^∧^); and significantly higher than NorLeu at 20, 30, 50, 60, 70, 90, 100, and 110 min (^t^). Further analysis was done on samples incubated with CellROX and *S. aureus* for 90 min and quantified by flow cytometry **(E)**. Schematic represents the different types of readouts from a plate reader compared to a flow cytometer in regards to CellROX assays **(F)**. Statistics was done using Prism 6 software ANOVA comparing all groups to saline treated *db/db*; ^*^p ≤ 0.05, ^**^p ≤ 0.01, ^***^p ≤ 0.001.

Bacteriocidal activity and PMN function were measured *ex-vivo* using whole blood assayed for *S. aureus* neutralizing activity. *S. aureus* CFUs were measured before inoculation and after a 30 min incubation with whole blood to calculate the percentage of surviving bacteria (Figure 2A). Blood from htz or A(1-7)-treated or NorLeu-treated diabetic animals had significantly lower bacteria levels than that from diabetic saline-treated mice. This suggests the reduced innate immune responses seen in diabetes is ameliorated by Mas agonist treatment.

### PMN Function Is Impaired in *db/db* Mouse Model of T2DM and Rescued With A(1-7) or NorLeu Treatment

To assess PMN function directly, whole blood from *htz* and *db/db* mice treated with saline, A(1-7) or NorLeu was incubated with *S. aureus* labeled with a fluorescent tag activated by the low pH in phagosomes (pHrodo) and read on fluorescent plate reader to determine the kinetic profile of phagocytosis (Figure 2B). Samples from the *htz* mice had the highest readout of fluorescence throughout the 110 min incubation, followed by the *db/db* mice treated with A(1-7) or NorLeu. Saline-treated *db/db* mice had the lowest emission curve; however, none of the changes in this assay were significant. In the next study, blood samples were collected, WBC were enumerated and incubated with *S. aureus*-pHrodo for 90 min, fixed and analyzed by flow cytometry. PMNs were gated by forward and side scatter and the % PMNs in total WBCs was measured (Supplemental Figures 1C,D). A calculation was then done to determine the number of total and phagocytic PMNs per mL of blood (Figure 2C). PMNs in the blood of saline-treated *db/db* mice were less phagocytic compared to the *htz* and A(1-7)- or NorLeu-treated diabetic animals.

### Immune Cell Activation Is Affected by T2D and Mas Activation

Cellular generation of reactive oxygen species (ROS) was measured in the whole blood in the absence and presence of *S. aureus*-pHrodo using CellROX dye (Figure 2D). In the absence of stimulation, blood from all three groups had a similar and constant baseline of CellROX reactivity with the *db/db* A(1-7) or NorLeu-treated mice showing the lowest emission level; no significant difference between the study groups was observed. With *S. aureus*-pHrodo stimulation, blood from all groups had an increase in CellROX reactivity over their unstimulated baselines and a constant increase in fluorescence throughout the 110 min incubation indicating persistent ROS generation. The blood from saline-treated *db/db* mice had the greatest increase in CellROX reactivity over baseline: significantly higher than htz at 50, 60, 70, 100, and 110 min; significantly higher than A(1-7) at 20, 30, 40, 50, 60, 70, 90, 100, and 110 min; and significantly higher than NorLeu at 20, 30, 50, 60, 70, 90, 100, and 110 min (Figure 2D). For flow cytometric analysis, blood samples were stimulated with *S. aureus*-pHrodo in the presence of CellROX for 90 min, washed, fixed and analyzed by flow cytometric analysis. PMNs were gated as distinct populations by forward and side scatter. Cellular activation in PMNs was measured by CellROX; activation was similar in both htz and saline-treated *db/db* mice. However, significantly higher activation was seen in A(1-7) and NorLeu-treated *db/db* mice (Figure 2E).

The differences seen between the plate reader and flow cytometry measurements of CellROX point to functional PMN changes. Samples from saline-treated *db/db* mice analyzed with the plate reader show the highest CellROX signal, but when analyzed by flow cytometry they have the lowest amount of CellROX signal. This suggests an increase in the production of extracellular ROS through degranulation of PMNs instead of internal pathogen degradation in saline-treated *db/db* mice (Figure 2F). Increases in the degranulation response of PMNs to bacterial challenge may partly explain the higher overall amount of tissue damage frequently seen in infected tissues of diabetic patients. Proper pathogen removal by phagocytosis as opposed to degranulation will reduce the time to clear the infections and reduce the amount of tissue damage (33).

### Overall PMN Health May Be Affected Early in Progenitor Development

**Figure 3:**
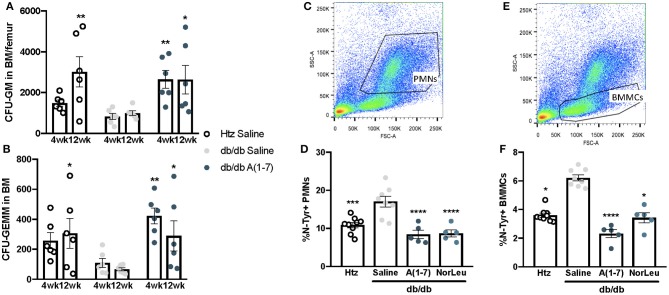
Neutrophil and monocyte progenitors are deficient in *db/db* mice and rescued by A(1-7) treatment. Neutrophils and monocytes have common progenitor lineage. BM was collected from *htz* and *db/db* mice treated with saline and A(1-7) at 0.5 mg/kg/day for 4, 8, and 12 weeks. Cells were cultivated in MethoCult™ media and both CFU-GEMMs **(A)** and CFU-GMs **(B)** were counted per well and multiplied by total number of WBCs in the BM of both femurs. BM cells were also stained with anti-N-Tyr antibody conjugated to FITC and quantified by flow cytometry. This set of animals included *htz* controls and *db/db* mice treated with saline or A(1-7) at 2 mg/kg/day. Cellular damage by OS was quantified in PMNs **(C,D)** and BMMCs **(E,F)** and expressed as percentage of cells positive for intracellular N-Tyr staining. Statistics was done using Prism 6 software ANOVA and compared to saline treated *db/db* mice; ^*^p ≤ 0.05, ^**^p ≤ 0.01, ^***^p ≤ 0.001, ^****^p ≤ 0.0001.

PMNs have a high turnover in the blood and are continuously sourced from the BM. Monocytes and neutrophils share common progenitor cells known as CFU-GM and its earlier progenitor CFU-GEMM. As the pathology of diabetes is believed to result from cumulative damage, we measured the CFU-GM and CFU-GEMM number in the BM was measured over time. Saline-treated diabetic animals consistently had lower numbers of both CFU-GEMM and CFU-GM, reaching significant difference from both *htz* and *db/db* A(1-7)-treated animals at 12 weeks of treatment (Figures 3A,B). OS was measured in the BM by FITC-anti-N-Tyr staining and quantified by flow cytometry. BM mononuclear cells (BMMCs) (Figures 3C,D) and PMNs (Figures 3E,F) were gated out by forward and side scatter. BMMCs have a lower SSC profile and mostly contain stem cells (CD45^−^Sca-1^+^) and other progenitor cells that have not yet started producing CD45 (Supplemental Figure 2); PMNs are appear higher than BMMCs in SSC and consist mostly Neutrophils (CD45+ Ly6G^+^Ly6C^lo^) and some Eosinophils (CD45^+^ F4/80^+^Siglec-F^+^). In both BMMCs and PMNs, the saline-treated diabetic animals had a significantly higher percentage of N-Tyr^+^ cells; A(1-7) or NorLeu-treated diabetic animals has levels similar to the *htz* group. This suggests an early effect, at the progenitor level, of T2D on PMN and monocyte health; possibly induced by increased OS. A(1-7) and NorLeu treatment may have therapeutic effects very early in innate immune cell development.

### Immunity Through Macrophages Is Also Impaired by Diabetic Conditions and Rescued by Mas Activation

**Figure 4:**
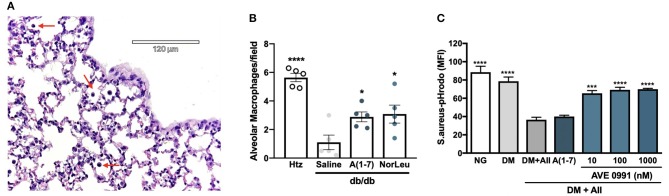
Diabetes altered macrophages respond to Mas-agonists *in vivo* and *in vitro*. Pictures were taken of H&E stained lung sections of *htz* and diabetic infection-free animals treated with saline or A(1-7) at 0.5 mg/kg/day. A representative picture from a *htz* mouse is shown with arrows pointing the AMs **(A)**. AMs were counted per image and an average per frame was calculated for each group **(B)**. *In vitro* assays were done to quantify the effect or DM and RAS-modification on the phagocytic capabilities of RAW-1 cells **(C)**. Cells were incubated in normal media (NG), media supplemented with 20 mM glucose, endothelin-1 and cortisol (DM), DM media with AII every 24 h (DM+A-II), and DM+A-II supplemented every 24 h with a Mas agonist. After 48 h in treatment media cells were harvested and incubated with *S. aureus*-pHrodo, results are shown as MFI for *N* = 5 per group; all groups were compared to (DM+AII). Statistics was done using Prism 6 software ANOVA; ^*^p ≤ 0.05, ^***^p ≤ 0.001, ^****^p ≤ 0.0001.

The two most common populations of macrophages in the respiratory tract are AMs and interstitial macrophages (IMs). Although there are molecular markers that are different between these 2 subtypes of pulmonary macrophages, their localization is the most consistent defining characteristic (34–36). In this study AMs, characterized by alveolar localization and morphology, were quantified in sections of lung tissue of non-infected *htz* and diabetic mice treated with saline, A(1-7) or NorLeu (Figure 4A). Saline-treated diabetic mice had significantly lower numbers of AMs than *htz* mice and A(1-7) or NorLeu-treated *db/db* mice, suggesting Mas activation protects AMs (Figure 4B). Because of their location, AMs are the first line of defense against many pathogens in the respiratory tract and, if depleted, can account for the delayed pathogen clearance and increase in pulmonary infections that are seen in diabetic patients and *db/db* infection model.

### Diabetic Conditions Induced *in vitro* Affect Macrophage Function and Protected by Mas Activation

RAW-1 cells have long been used to test for *in vitro* efficacy of novel therapeutics because of their superior consistency in phagocytic assays (37). Here, RAW-1 cells were treated with various media conditions for 48 h and then incubated with *S. aureus*-pHrodo to determine their ability to phagocytose pathogens (Figure 4C). Cell incubated with DM show little decrease in phagocytic capabilities unless AII is included. A(1-7) treatment did not rescue this phenotype, possibly because of its short half-life in media supplemented with FBS. AVE 0991, a small molecule Mas agonist, treatment of RAW-1 cells incubated in DM+AII restored the ability of the RAW-1 cells to phagocytose *S. aureus*. Mas activation protected against suppression of phagocytosis in a cell involved in innate immunity in cells exposed to this physiologically relevant diabetic culture medium, further defining Mas as a therapeutic target for diabetic immunosuppression.

## Discussion

Pulmonary infections are a common and significant threat to patients with diabetes. A combination of an impaired immune system and tissue damage increases the chance of a severe complication (38, 39). Diminished innate immune function can cause uncontrolled bacterial growth and lead to pneumonia or death (40). Current therapies for T2DM focus on lowering blood glucose, insulin sensitization and weight loss. These treatments are widely used by diabetic patients and, in many cases, effectively lower HbA1c levels; however, many of the diabetic co-morbidities persist despite blood glucose normalization perhaps due to continued increased OS leading to chronic inflammation (4, 6). Beyond glycemic control, treatments for diabetic patients need to also focus on the underlying cause of all complications to mitigate the consequences.

Neutrophil degranulation and LPS-induced immune activation have been shown to be negatively affected by hyperglycemia. Leukocytes increase production of ROS in diabetic patients, possibly due to the hyperglycemic environment (41, 42). Although hyperglycemia, ROS and inflammation can all affect cell function, existing compounds that decrease ROS levels and inflammation can benefit the patient (4–7, 43). Part of the underlying cellular pathological in T2DM is associated with the chronic up-regulation of the pathological arm of the RAS. AII is the main active peptide in this pathological arm and, through binding to AT1, cellular functions are altered leading to an increase in ROS and inflammation. In diabetic patients, AII is known to be upregulated potentially leading to the tissue damage seen in diabetes-related complications (9). Our studies here show that diabetic milieu, including high glucose, cortisol and endothelin 1, is not enough to reduce macrophage phagocytosis *in vitro*, AII is required; further supporting its key role in the diabetic environment that hinders proper immune function. To decrease the burden of these co-morbidities on both the patient and the health care system, the root cause of the immune dysfunctions and therapies needs to be identified and addressed.

Previous studies as well as those presented here suggest that diabetes impacts innate immunity by hindering the activity of PMNs. Changes in phagocytic capabilities of the PMNs correlated with changes in the ability of PMNs to kill bacteria. Macrophage health is also affected by diabetic conditions. Progenitors to both populations are not only lower in number in diabetic animals, they also exhibit increased OS. All of these pathological changes as a result of diabetes were not observed in A(1-7) or NorLeu-treated *db/db* mice despite the absence of glucose control. Evidence suggests that this is partly due to reduced OS, a parameter not currently targeted by diabetic medications. Treatment with A(1-7) or NorLeu reduced OS and can counteract the pathological effects of AII in various other disease models and is known to have distinct actions according to tissue and pathology (14, 15, 43, 44).

We also noted a shift in cellular response to pathogens in saline-treated diabetic animals compared to the *htz* animals. After inoculation with *S. aureus*, in the samples from saline-treated *db/db* mice, ROS was increased in the extracellular environment. Conversely, in *htz* and A(1-7) or NorLeu-treated *db/db* mice, these mediators were increased in the intracellular compartments. Since the phagocytic function of PMNs is compromised in diabetes, it may be that the cells are trying to clear pathogens by producing a ROS-rich environment at the site of infection. A(1-7) and NorLeu-treated animals did not share this phenotype, they primarily act through phagocytosis to neutralize bacteria, despite their diabetic status. Focus on extracellular ROS production may help explain the higher amounts of tissue damage seen in T2DM patients since ROS will damage surrounding tissues along with invading pathogens. This neutrophil dysfunction may also be the reason for increased severity of infections (45, 46) and delayed wound healing seen in diabetic patients (47, 48). Our results are also consistent with other findings that suggest that there is BM dysfunction in diabetic patients (49). Although, A(1-7) has been shown to decrease inflammation in previous studies (50), our observations were seen in the absence of changes in circulating cytokines, suggesting a direct impact on PMN activity directly impacting tissue health. The exact molecular mechanism by which A(1-7) acts seems to be dependent on the cell type and insult to the system (50).However, previous studies suggest that the anti-inflammatory actions of RAS-modification are likely mediated by inhibition of NF-kB (51, 52) or MAPK (53) signaling pathways.

Taken together, the data presented indicate that RAS-modification is a viable therapeutic target for the treatment of diabetic complications, specifically those affected by inadequate innate immune function. Further, these studies show that despite the short half-life of A(1-7) and NorLeu we can measure changes in innate immunity with once daily dosing. A recent pharmacoeconomic analysis of the effect of inhibitors of the pathological of RAS on pulmonary infections supporting the translatability of these results to diabetic patients. Using a de-identified insurance claims data set it was shown that even newly diagnosed T2DM is associated with higher incidence of pulmonary infections and that RAS-modifying drugs can reduce these outcomes (54). Future studies will explore mechanisms by which Mas agonism improves immune function in diabetic patients and the role of other immune cells in this paradigm. Ideally, an orally available Mas agonist that replicates the actions of A(1-7) can be developed as a therapeutic to help improve the patient health in T2DM.

## Data Availability Statement

The datasets generated for this study are available on request to the corresponding author.

## Ethics Statement

The animal study was reviewed and approved by the University of Southern California Institutional Animal Care and Use Committee.

## Author Contributions

All of the authors contributed significantly to the work presented here. MS and KR contributed to the experiment planning, and interpretation of data. MS and KG contributed to the execution of experiments. MS wrote the manuscript. KR and KG edited the manuscript.

### Conflict of Interest

The authors declare that the research was conducted in the absence of any commercial or financial relationships that could be construed as a potential conflict of interest.
