# Supplementary material for: Improving the Innate Immune Response in Diabetes by Modifying the Renin Angiotensin System
Source: Front Immunol. 2019 Dec 10;10:2885. doi: 10.3389/fimmu.2019.02885 (PMC6914815; doi:10.3389/fimmu.2019.02885)
Supplement: Supplementary file 1 [file Data_Sheet_1.docx]

Supplementary Material

## Supplementary Figures

**C**

**D**

**A**

**B**

**Supplemental Figure 1.** Weight, cytokine profile and PMN numbers are not significantly affected by treatment with Mas agonists in 16wk old mice. Eight week-old mice were treated with saline (htz and db/db), A(1-7) at 0.5mg/kg/day (db/db), or NorLeu at 0.5mg/kd/day (db/db) for 8 weeks. At the end of treatment mice were weighed (A) and plasma was collected before and after infection with *S. aureus*. Plasma was used to measure levels of circulating TNF-a, IL-6, and IL-10 (B). The %of PMNs (C) total number of PMNs/ml of blood (D) were measured using flow cytometry. Statistics was done using Prism 6 software ANOVA and compared to saline treated db/db mice; ****p ≤ 0.0001.

**Supplemental Figure 2. Gating strategy used for BM Characterization.** BM from WT, 12 week old mice was isolated, stained and analyzed by flowcytometry. FSC-A vs FSC-H was used to determine singlets, and live cells were distinguished using DAPI. Neutrophils (blue) were characterized as CD45^+^, Ly6G^+^ and Ly6C^lo^, and generally appear at the higher SSC population. Eosinophils (red) were characterized as CD45^+^, F4/80^+^ and Siglec-F^+^, and also appear at the higher SSC population. The CD45- population is likely made up of stem and progenitor cells, here we stained for Sca-1 (green) as a general stem cell marker, these cells appear in the lower SSC population.
